# Structural Network Efficiency Predicts Resilience to Cognitive Decline in Elderly at Risk for Alzheimer’s Disease

**DOI:** 10.3389/fnagi.2021.637002

**Published:** 2021-02-22

**Authors:** Florian U. Fischer, Dominik Wolf, Oliver Tüscher, Andreas Fellgiebel, on behalf of Alzheimer’s Disease Neuroimaging Initiative

**Affiliations:** ^1^Department of Psychiatry and Psychotherapy, University Medical Center Mainz, Johannes Gutenberg University Mainz, Mainz, Germany; ^2^Center for Mental Health in Old Age, Landeskrankenhaus (AöR), Mainz, Germany; ^3^Leibniz Institute for Resilience Research (LIR), Mainz, Germany

**Keywords:** resilience, structural network, connectome, Alzheimer’s disease, amyloid, white matter, diffusion imaging

## Abstract

**Introduction**: Functional imaging studies have demonstrated the recruitment of additional neural resources as a possible mechanism to compensate for age and Alzheimer’s disease (AD)-related cerebral pathology, the efficacy of which is potentially modulated by underlying structural network connectivity. Additionally, structural network efficiency (SNE) is associated with intelligence across the lifespan, which is a known factor for resilience to cognitive decline. We hypothesized that SNE may be a surrogate of the physiological basis of resilience to cognitive decline in elderly persons without dementia and with age- and AD-related cerebral pathology.**Methods**: We included 85 cognitively normal elderly subjects or mild cognitive impairment (MCI) patients submitted to baseline diffusion imaging, liquor specimens, amyloid-PET and longitudinal cognitive assessments. SNE was calculated from baseline MRI scans using fiber tractography and graph theory. Mixed linear effects models were estimated to investigate the association of higher resilience to cognitive decline with higher SNE and the modulation of this association by increased cerebral amyloid, liquor tau or WMHV. **Results**: For the majority of cognitive outcome measures, higher SNE was associated with higher resilience to cognitive decline (*p*-values: 0.011–0.039). Additionally, subjects with higher SNE showed more resilience to cognitive decline at higher cerebral amyloid burden (*p*-values: <0.001–0.036) and lower tau levels (*p*-values: 0.002–0.015).**Conclusion**: These results suggest that SNE to some extent may quantify the physiological basis of resilience to cognitive decline most effective at the earliest stages of AD, namely at increased amyloid burden and before increased tauopathy.

## Introduction

*In-vivo* amyloid imaging has profoundly improved the diagnosis of Alzheimer’s disease (AD) at its pre-dementia stages. However, individual predictions of cognitive decline are unsatisfactory from a clinical perspective due to considerable variance (Vos et al., 2013; Insel et al., 2016; Jack et al., 2016; Donohue et al., 2017), which presumably refers to an individual’s capacity to tolerate or compensate cerebral pathology, commonly termed reserve or—more generally—resilience (Yaffe et al., 2011; Barulli and Stern, 2013; Cabeza et al., 2018; Wolf et al., 2018). The identification and quantification of an MRI-based surrogate of the physiological basis of this resilience could thus complement and significantly improve individual predictions of cognitive decline based on cerebral pathology. However, the underlying physiological basis of resilience to cognitive decline has not conclusively been identified so far.

For an extensive discussion of hypotheses, please refer to Barulli and Stern (2013) and Cabeza et al. (2018). Briefly, functional imaging studies have demonstrated that sustained cognition in aging is associated with maintained functional connectivity (Tsvetanov et al., 2016, 2018). Furthermore, in higher age and in the presence of cerebral pathology, the brain seems to recruit more neural resources for given cognitive tasks as compared to younger subjects or those with less pathology present, which may be a resilience mechanism (Sebastian et al., 2013; Reuter-Lorenz and Park, 2014; Stargardt et al., 2015; Fernández-Cabello et al., 2016).

As functional imaging is limited to the specific tasks or situations of the experiment performed, it is complementary to these experiments to investigate the brain structures underlying brain functions, whose structural organization demonstrably coincides with and possibly modulates functional connectivity (Damoiseaux, 2017). However, rather than being specific to tasks brain structure is arguably the product of a lifetime’s individual cognitive profile and thus reflects a convolute of general everyday cognition. This notion is supported by the repeated finding of the association of the brain’s structural organization and intelligence (Li et al., 2009; Fischer et al., 2014; Bathelt et al., 2018; Koenis et al., 2018), which aims to measure the underlying general factor of cognitive performance across different cognitive domains and tasks (Deary et al., 2010). Interestingly, intelligence is also a known resilience factor to cognitive decline (Schmand et al., 1997; Whalley et al., 2004; Stern, 2012). These findings led us to hypothesize that it may be worthwhile to investigate the brain’s structural connectome as a potential predictor for resilience to cognitive decline. In light of the repeatedly demonstrated associations with intelligence cited above (Li et al., 2009; Fischer et al., 2014), we chose global efficiency of the network constructed from GM segments and reconstructed WM connections, a measure that aims to model and quantify parallel information transfer capacity (Li et al., 2009), for the present study over the myriad of other available graph theory-based options.

## Materials and Methods

Data used in the preparation of this article were obtained from the Alzheimer’s Disease Neuroimaging Initiative (ADNI) database^1^. The ADNI was launched in 2003 as a public-private partnership, led by Principal Investigator Michael W. Weiner, MD. The primary goal of ADNI has been to test whether serial magnetic resonance imaging (MRI), positron emission tomography (PET), other biological markers, and clinical and neuropsychological assessment can be combined to measure the progression of mild cognitive impairment (MCI) and early Alzheimer’s disease. For up-to-date information, see www.adni-info.org. All procedures followed were as per the ethical standards of the responsible committee on human experimentation (institutional and national) and with the Helsinki Declaration of 1975, and the applicable revisions at the time of the investigation.

### Subjects

Subjects and their respective data points were selected from the database of the ADNI project according to the following criteria: enrollment during the ADNI 2 phase in the cognitively normal or MCI group, availability of two or more longitudinal neuropsychological assessments as well as the availability of the following imaging data at baseline: T1, FLAIR, diffusion-weighted (DWI) MRI and florbetapir amyloid (AV45) PET. MCI subjects were included to ensure sufficient variance of cognition and pathological markers in a continuum of elderly non-demented subjects for the assessment of possible resilience. For details regarding the cognitive assessment within the ADNI, please refer to the publicly available procedures manual https://adni.loni.usc.edu/wp-content/uploads/2008/07/adni2-procedures-manual.pdf. In total, 85 subjects consisting of 34 females and 51 males aged 56.5 to 89.0 years were included (see Table 1 for demographics). Informed consent was obtained from all subjects for being included in the study.

**Table 1:** Sample demographics and descriptive statistics–mean ± standard deviation.

	CN	MCI	Total group	*p*-value
*N*	34	51	85	
Gender F/M	18/16	16/35	34/51	0.078
Age at baseline	73.4,6.4	72.1,6.6	72.6,6.5	0.651
Education	16.4,2.6	16.0,2.7	16.1,2.7	0.500
APOE4 +/−	25/9	16/35	41/44	<0.001^*
ADAS-cog	8.5,3.9	18.6,7.13	14.5,7.8	<0.001^*
CDR-SOB	0.04,0.14	1.45,0.81	0.89,0.94	<0.001^*
MMSE	28.9,1.4	27.6,1.8	28.1,1.8	<0.001^*
Network efficiency	252.9,18.9	249.3,15.4	250.6,16.8	0.217
AV45-PET	1.08,0.14	1.26,0.24	1.19,0.22	0.001^*
CSF TAU	61.29,23.98	98.30,61.02	83.50,52.68	0.004^*
WMHV	0.38,0.28	0.58,57	0.53,0.49	0.397

CN, cognitively normal; MCI, mild cognitive impairment. *P*-value of group differences as calculated by Mann–Whitney test for continuous and Pearsons’s Chi-squared test for categorical variables. Education, in years. APOE4: apolipoprotein ɛ4, the positivity of one or two alleles. ADAS-cog, Alzheimer’s disease assessment scale; CDR-SOB, Clinical dementia rating sum of boxes; MMSE, minimental state exam; AV45-PET: florbetapir positron emission tomography, global florbetapir standardized uptake value ratio. CSF TAU: liquor specimen total tau aliquot. WMHV: normalized white matter hyperintensity volume, percent. *Statistically significant.

### Corticospinal Fluid Measurement

All corticospinal fluid (CSF) biomarkers collected at different centers were stored and analyzed at the Penn ADNI Biomarker Core Laboratory at the University of Pennsylvania, Philadelphia, PA, USA. CSF concentrations of total tau were measured in the baseline CSF samples using the multiplex xMAP Luminex platform (Luminex Corporation, Austin, TX, USA). We included total tau instead of phosphorylated tau, as the latter is included in the former and total tau thus includes tauopathy specific to AD as well as more general tauopathy that is potentially relevant for a cognitive outcome (Blennow et al., 2001). More details on data collection and processing of the CSF samples can be found elsewhere^2^ (Shaw et al., 2009).

### APOE Genotype

Apolipoprotein (APOE) genotype was determined by genotyping the two single nucleotide polymorphisms that define the APOE ε2, ε3, and ε4 alleles (rs429358, rs7412) with DNA extracted by Cogenics from a 3-ml aliquot of EDTA blood^3^.

### Imaging Data Acquisition

DWI, FLAIR, and inversion-recovery spoiled gradient recalled (IR-SPGR) T1-weighted imaging data were acquired on several General Electric 3T scanners using scanner specific protocols. Briefly, DWI data was acquired with a voxel size of 1.37^2^ × 2.70 mm^3^, 41 diffusion gradients, and a *b*-value of 1,000 s/mm^2^ using an echo-planar imaging sequence with a 90° flip angle. T1-weighted data were acquired using a gradient-echo sequence with an 11° flip angle and a voxel size of 1.02^2^ × 1.20 mm^3^.

AV45 PET imaging data were acquired on several types of scanners using different acquisition protocols. To increase data uniformity, the data underwent a standardized preprocessing procedure at the ADNI project. All imaging protocols and preprocessing procedures are available at the ADNI website^2^.

### T1-Weighted and FLAIR Data Processing

The T1-weighted IR-SPGR data were automatically tissue-segmented and spatially normalized to MNI-space using SPM8^4^ and the VBM8-toolbox^5^. Additionally, inverse transformations from MNI to native T1 space were calculated.

Gray matter (GM) was segmented into 106 functionally and anatomically defined cortical regions, as well as the subcortical basal ganglia regions as implemented in the probabilistic Harvard Oxford Atlas, supplied with FSL.

Total intracranial volume (TIV), as well as hyperintensity volume (WMHV), were calculated at ADNI core laboratories from T1-weighted and FLAIR data using published tissue segmentation methods (DeCarli et al., 2005; Fletcher et al., 2012). WMHV was also normalized by dividing by the TIV.

### DWI Data Processing

DWI data were corrected for eddy currents and motion artifacts using the method of Rohde et al. (2004) as implemented in VistaSoft; diffusion gradients were adjusted according to the resulting transformations. Additionally, DWI data were upsampled to 1 mm isotropic voxel size for further processing. For fiber tractography, Anatomically Constrained Tractography (ACT) as implemented in MRtrix was employed (Smith et al., 2012). This approach incorporates anatomical constraints based on tissue segmentation of T1 data. To this end, VBM8 tissue segmented data in T1 native space were coregistered using SPM8 to the upsampled DWI B0 images and used in the subsequent ACT. Based on the tissue segmentation images, the ACT framework calculates an isocontour representing the interface of GM and WM for fiber seeding. Subsequently, tractography seed points were placed randomly along the GM-WM interface. Starting from these points, the probabilistic “ifod2” tractography algorithm was executed until 500,000 anatomically plausible streamlines were reconstructed for each subject. Streamlines were accepted if they met the anatomical constraints of ACT (Smith et al., 2012).

### Network Reconstruction and Characterization

To reconstruct fibers, the 106 Harvard Oxford Atlas GM ROIs were first warped to native T1 space using the inverse VBM8 normalization transformations and then transferred to the upsampled DWI space using the transformation estimated from the T1 to B0 coregistration.

Subsequently, for each ROI pair in each subject, the number of the previously reconstructed streamlines (see above) intersecting both ROIs was obtained and recorded to construct the adjacency matrix. A connection threshold of at least three connecting streamlines for each connection was applied (Li et al., 2009; Fischer et al., 2014). Finally, structural network efficiency (SNE) for each subject was calculated as the average efficiency of the network according to the formula

$$SNE=\text{1}/\left(n*\left(n-\text{1}\right)\right)*Sum_{i\neq j}\left(\text{1}/d_{ij}\right)$$

where *n* represents the number of nodes and *d_ij_* is the inverse of the connection weights of the shortest path between node *i* and *j* (Latora and Marchiori, 2001). For a detailed description and discussion of the graph measures, see Fornito et al. (2015).

### PET Data Processing

Subjects’ global cortical amyloid-β load was calculated from AV45 PET images according to procedures established by the ADNI^6^. Briefly, cortical amyloid was calculated as the average of the AV45 uptake in the frontal, angular/posterior cingulate, lateral parietal, and temporal cortices normalized by dividing by the mean uptake in the cerebellum.

### Neuropsychological Assessment

Subjects underwent an extensive longitudinal neuropsychological assessment generally every 12 months over a span of years varying individually with the date of inclusion in the study. For details regarding the cognitive assessment within the ADNI, please refer to the publicly available procedures manual^7^. Within the scope of this study, the cognitive Alzheimer’s Disease Assessment Scale (ADAS-cog), which spans several cognitive domains (Rosen et al., 1984), as well as the Clinical Dementia Rating Scale Sum of Boxes (CDRSOB) and the Mini-mental State Examination (MMSE) were investigated. See Table 1 for descriptive statistics.

### Resilience Assessment Model

In the present article, resilience to cognitive decline is modeled employing a tetrad of sets of

(i) longitudinal measures quantifying cognitive outcome (COG)—specifically ADAS-cog, CDRSOB, and MMSE,
(ii) measures of age-associated cerebral pathology (PATH)—specifically AV45, TAU, and WMHV at baseline,
(iii) a candidate resilience factor at baseline (RES)—specifically SNE, and
(iv) a measure of time relative to baseline (T)—specifically the number of months from baseline where the respective cognitive assessments were performed.

These are combined in a general linear model in the following way:

COG ~ T + PATH + RES + T * PATH + T * RES + T * RES * PATH + RES * PATH

In older adults, the cognitive outcome may typically decline over time, reflected in a negative association of COG and T. Furthermore, pathology may additionally negatively modulate this association, reflected by a negative association of T*PATH with COG. However, potential resilience factors may contribute to a slower decline of cognitive outcome over time, reflected by a positive association of T*RES with COG. More specifically, the terms of interest and their interpretations within the scope of this study were:

T*RES: the modulation of the time-dependent change of COG by the candidate resilience factor (RES) independent of present PATH → if positive, this is termed **general resilience to cognitive decline**

T*RES*PATH: the modulation of the time-dependent change of COG by the candidate resilience factor (RES) that is dependent on present PATH → if positive, this is termed **dynamic resilience to cognitive decline**; if negative, this is termed l**imited resilience to cognitive decline**.

This model is based on a previously published statistical approach to comprehensively quantify resilience (Wolf et al., 2018; Fischer et al., 2019) that has been extended here to accommodate longitudinal neuropsychological data akin to (Donohue et al., 2017). Additionally, a more comprehensive description is included in the Supplementary Material to this article.

### Statistical Analysis

Descriptive statistics, as well as differences between the CN and MCI groups, were calculated for all measures included in subsequent statistical analyses using Mann–Whitney tests for continuous and Pearson’s Chi-squared test for categorical variables.

The resilience assessment model described above was implemented using linear mixed-effects regressions that included age, gender, years of education, and APOE4 positivity as covariates as well as random effects for the subject and clinical status (i.e., CN and MCI). For each cognitive measure, a group of models with and without the model terms for general resilience as well as dynamic/limited resilience for measures of pathology (amyloid, tau WMHV) were estimated. Within each group, the model with the lowest conditional AIC (cAIC; Greven and Kneib, 2010) was regarded as best explaining the data. All models with a conditional AIC exceeding the cAIC of the best model within that group by less than 2 (i.e. Δ cAIC < 2) were regarded as having substantial evidence. The other models were discarded. Additionally, marginal *R*^2^ was calculated for these models (Nakagawa and Schielzeth, 2013). To lessen the chance of overfitting, significance testing was then conducted only on the resilience terms contained in all models retained within the respective group, by using the likelihood-ratio test (LRT) and additionally parametric bootstrapping at 1 mio. simulations.

All statistical analyses were conducted using R 3.4.0 as well as the packages “lme4” (Bates et al., 2014), “robustlmm” (Koller, 2016), “cAIC4” (Säfken et al., 2018), “pbkrtest” (Halekoh and Højsgaard, 2014) as well as “car” (Fox and Weisberg, 2019). Variance inflation was calculated for the best model for each cognitive outcome variable. ADAS-cog was inverted such that higher values meant better performance. WMHV was log-transformed to achieve approximate normal distribution. The significance threshold was set to *α* = 0.05 for all analyses. Results of the resilience models were corrected for FDR at 5%. A more technically comprehensive description of the statistical analysis with a listing of all models estimated can be found in the Supplementary Material to this article.

## Results

The following variables showed significant differences between the CN and MCI groups: APOE4 positivity (*p* < 0.001), AV45 (*p* < 0.001), TAU (*p*: 0.004), ADAS-cog (*p* < 0.001), CDRSOB (*p* < 0.001) and MMSE (*p* < 0.001). For descriptive statistics of all variables considered, please refer to Table 1.

All mixed-effects regression models that best explained the data for each cognitive measure according to cAIC contained the term for general resilience (T*SNE) as well as a term for dynamic resilience at elevated amyloid (T*SNE*AV45) and limited resilience at elevated tau levels (T*SNE*TAU). Additionally, the best model for CDR-SOB contained a term for limited resilience at elevated WMHV (T*SNE*WMHV). The marginal *R*^2^ for the groups of models that best explained the data ranged from 0.405 to 0.433. Please refer to the Supplementary Material for a list of the best models and associated statistical measures.

LRT yielded significant results for dynamic resilience at elevated amyloid (*p* < 0.00001–0.03549, standardized regression coefficients: 0.024–0.276) as well as for limited resilience at elevated tau (*p*: 0.00164–0.01535, standardized regression coefficents: −0.030 to −0.074) for all cognitive measures. Additionally, the general resilience term was significant for CDR-SOB and MMSE (*p*: 0.01138–0.03848, standardized regression coefficents: 0.074–0.098). Finally, the limited resilience term for elevated WMHV was significant only for CDR-SOB (*p*: 0.01433, standardized regression coefficient: −0.058).

**Figure 1:**
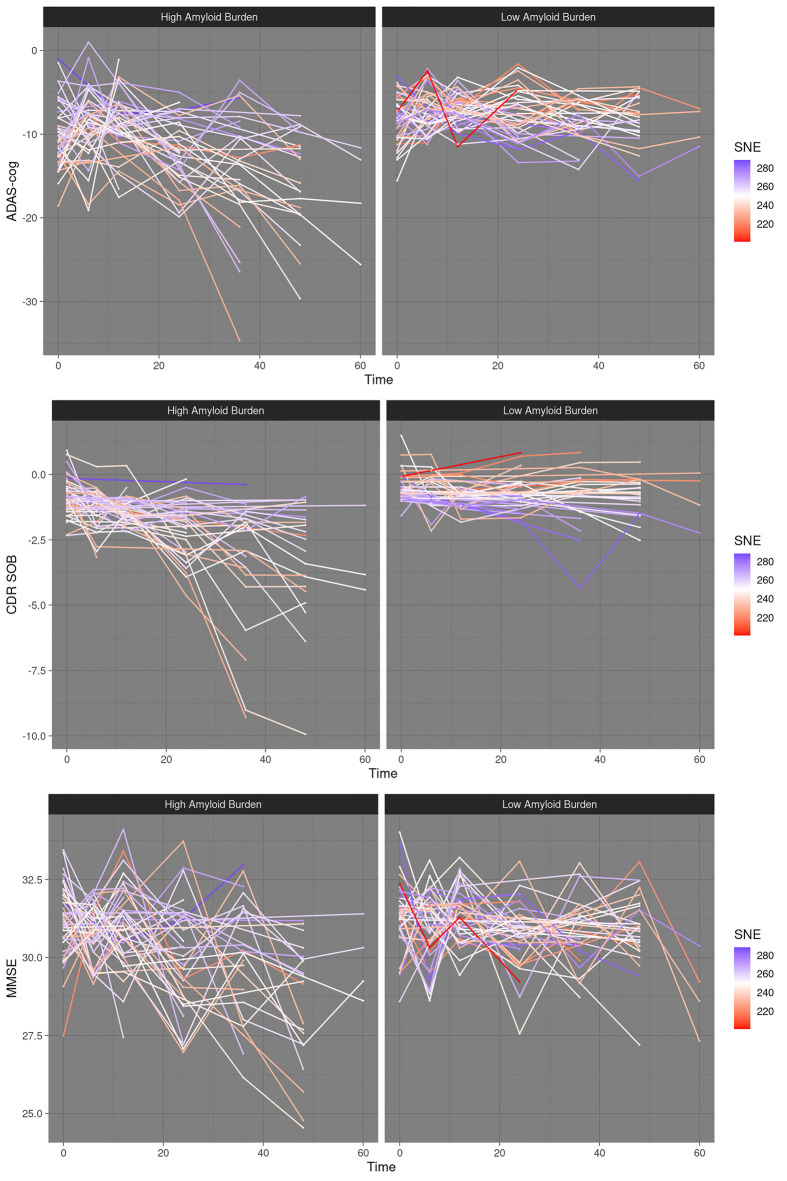
Dynamic resilience to cognitive decline at high amyloid burden–scatter plots. High amyloid burden vs. low amyloid burden, median split. SNE, structural network efficiency. Time, time in months after baseline. ADAS-cog, Alzheimer’s disease assessment scale; CDRSOB, clinical dementia rating sum of boxes; MMSE, mini-mental state examination.

**Figure 2:**
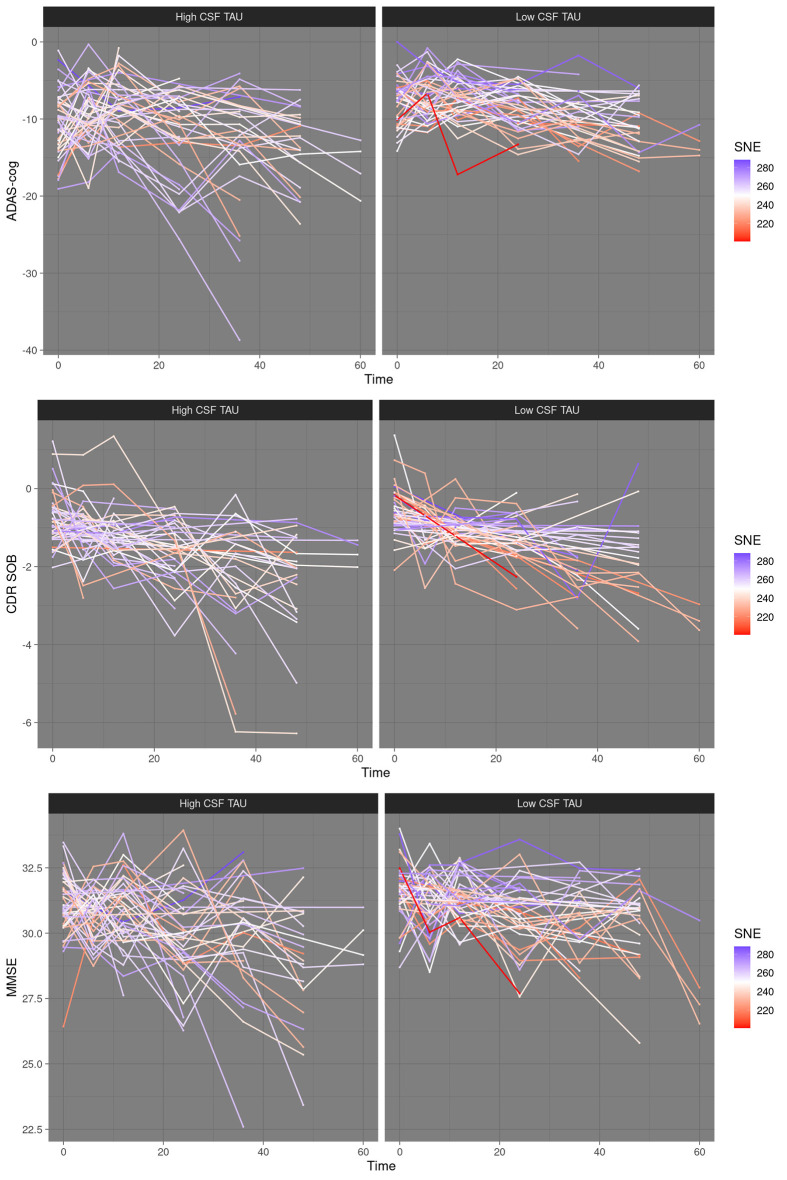
Limited resilience to cognitive decline at low corticospinal fluid (CSF) tau—scatter plots. High CSF tau vs. low CSF tau, median split. SNE, structural network efficiency. Time, time in months after baseline. ADAS-cog, Alzheimer’s disease assessment scale; CDRSOB, clinical dementia rating sum of boxes; MMSE, mini-mental state examination.

Robust reestimation of models yielded coefficients of the same directionality and comparable magnitude. FDR correction at 5% did not lead to the rejection of any of the results. *P*-values reestimated using parametric bootstrapping were similar to those calculated by LRT. Variance inflation factors were below two for all predictors in the best fitting models. For an overview of estimated coefficients and respective statistics please refer to Table 2. For scatter plots of the data points please refer to Figures 1, 2.

**Table 2:** Estimates of model terms of interest.

Cog Out	Term of interest	Std beta	Rob std beta	Chi^2^	*p*-value
ADAS-cog	T * SNE	–0.004	–0.009	0.044	0.83338
ADAS-cog	T * SNE * AV45	0.088	0.066	8.020	0.00463*
ADAS-cog	T * SNE * TAU	–0.074	–0.057	8.088	0.00446*
CDRSOB	T * SNE	0.098	0.090	4.284	0.03848*
CDRSOB	T * SNE * AV45	0.276	0.216	56.736	<0.00001*
CDRSOB	T * SNE * TAU	–0.069	–0.073	5.876	0.01535*
CDRSOB	T * SNE * WMHV	–0.058	–0.046	5.997	0.01433*
MMSE	T * SNE	0.074	0.085	6.405	0.01138*
MMSE	T * SNE * AV45	0.024	0.026	4.422	0.03549*
MMSE	T * SNE * TAU	–0.030	–0.026	9.914	0.00164*

Cog Out, Cognitive outcome; Std beta, standardized regression coefficient; Rob std beta, standardized regression coefficients of robust mixed-effects regression; ADAS-cog, Alzheimer’s disease assessment scale; CDRSOB, clinical dementia rating sum of boxes; MMSE, mini-mental state examination; SNE, *global efficiency of structural networks of reconstructed white matter tracts connecting segmented gray matter regions*. *Statistically significant.

## Discussion

The main results of this study indicate that in a population of non-demented elderly with varying amounts of age- and AD-related cerebral pathology, higher efficiency of the cerebral network may be associated with more resilience to cognitive decline. This association was increased at higher amounts of cerebral amyloid burden and decreased at higher levels of CSF tau. SNE may therefore be a factor of dynamic resilience to cognitive decline concerning amyloid load whilst being a limited resilience factor concerning tau burden.

To our best knowledge, this is the first study investigating the association of structural network properties and resilience to cognitive decline, quantified as lower cognitive decline unexplained by baseline cerebral pathology. However, other studies have demonstrated associations between WM network properties and other non-physiological resilience factors. Specifically, three studies demonstrated an association between intelligence and SNE (Li et al., 2009; Fischer et al., 2014; Bathelt et al., 2018). A fourth study demonstrated an association between education and network flow, a measure quantifying hypothetical rerouting capabilities of the network (Wook Yoo et al., 2015). Although these studies are of limited comparability due to differences in methodology and sample composition, one may be tempted to speculate that structural network properties and SNE specifically may form part of the physiological basis of intelligence and education as resilience factors. However, as education was controlled for in all analyses of the present study, the association of SNE with resilience to cognitive decline seems to go beyond the effects of education.

A hypothetical mechanism by which SNE may be associated with resilience to cognitive decline can be derived from its association with intelligence. Intelligence ratings consist of several cognitively demanding tests (Deary et al., 2010). The synchronized processing of these tasks by functional networks of distributed regions requires efficient and effective information transfer between them, which in turn depends on the structural connectivity and integrity of WM tracts (Penke et al., 2012). Additionally, there is evidence from functional imaging studies demonstrating that maintained functional connectivity is more associated with cognitive performance at higher age (Tsvetanov et al., 2016, 2018) and that the brain engages in a compensatory activity in aging and the presence of cerebral neuropathology by recruiting additional neural resources across the brain (Reuter-Lorenz and Park, 2014; Stargardt et al., 2015), which possibly depends likewise on structural integrity and efficient organization as a modulator of functional reorganization. Assuming that the brain’s compensatory reaction to age-associated pathological changes is alike to the compensatory reaction to aging, the findings of the present study could be explained as follows: as the aging brain accumulates deteriorative changes such as loss of GM volume and in some cases AD-related pathology such as amyloid accumulation, it engages compensatory processes aimed at recruiting more neural resources, whose recruitment for task processing depends upon information transfer efficiency, a surrogate measure of which may be SNE. It follows that as the brain accumulates age- and, potentially, AD-related deteriorative changes with time relative to baseline, SNE will become more important for sustained cognition and thus more associated with resilience. This is supported by the finding of significant positive interaction terms of the time variable with baseline SNE estimated for two out of three cognitive outcome measures considered.

Interestingly, the association of SNE with resilience to cognitive decline described above was increased in subjects with higher baseline cerebral amyloid load, thus indicating resilience to cognitive decline that is dynamic with respect to the amyloid burden. This result was consistent across all cognitive outcome measures investigated and is also consistent with the resilience mechanism proposed above: elevated cerebral amyloid load has been demonstrated to be associated with a higher rate of cognitive decline (Donohue et al., 2017). However, it is not locally associated with GM atrophy at the earliest preclinical stages of the AD trajectory (Karran et al., 2011; Kljajevic et al., 2014). In this scenario, compensation by recruitment of additional neural resources *via* the WM network would be both necessary as well as effective, as the to be recruited neural resources in the form of GM regions remain mostly intact. However, the opposite may be true for increased levels of CSF tau at baseline, where the decrease of cognitive decline due to higher baseline SNE was consistently lower for all three measures of cognitive outcome thus indicating resilience to cognitive decline that is limited concerning tau. Increased tau is usually accompanied by more clinically relevant cognitive impairment and neurodegeneration (Solé-Padullés et al., 2011; Amlien et al., 2013) especially at later stages of the AD-typical trajectory that is commonly referred to as the amyloid cascade (Jack et al., 2013). In this scenario, the to be recruited additional neural resources may already be impaired, which would probably render the compensatory functional reorganization less effective.

An alternative or perhaps a complementary explanation of the limited resilience to cognitive decline at increased levels of baseline CSF-tau is provided by the finding of WM microstructural integrity deterioration at increased levels of CSF-tau (Amlien et al., 2013). These may not be reflected in the SNE measure considered in this study, but impair network function such that compensation *via* recruitment of additional neural resources is rendered less effective (Fernández-Cabello et al., 2016). The same explanation could apply to the finding of limited resilience to cognitive decline at higher baseline volume of WMHV, which are associated with deterioration in local WM integrity and cognition (Vernooij et al., 2009).

In our view, the hypotheses put forward regarding limited resilience at increased tau warrant further studies investigating how tau-related GM and WM changes impact SNE. Additionally, as the tau measure employed in this study is a convolute of highly AD-specific hyperphosphorylated tau and other forms of tau that are also associated with other neurological disorders (Blennow et al., 2001; Skillbäck et al., 2015), their differential effects on SNE are of interest for the potential of SNE to act as a resilience factor at neuropathologically more advanced stages of AD.

When considering the points discussed, one ought to bear in mind that any variance of SNE associated with AV45, TAU, or WMHV was partialled out for the estimation of all other model coefficients. This may seem contradictory to the points made in the previous paragraph, where it was argued that TAU and WMHV may deterioratively affect the network and thus impair its provision of compensatory capabilities, which seems likely at least for elevated WMHV. This argument implies that the putative effects of tau and WMHV on the network are reflected in the SNE measure. If this had been the case, however, neither tau nor WMHV would have modulated the association of SNE with resilience to cognitive decline. To improve SNE as a surrogate measure of the network’s potential to support resilience, future studies might investigate modifications of SNE such that likely changes due to tau and WMHV as demonstrated previously (Vernooij et al., 2009; Amlien et al., 2013) will be reflected within a modified SNE measure.

Finally, apart from the mechanistic considerations discussed above, we believe that the results of this study may hold potential clinical relevance. The inclusion of SNE in the best models for all cognitive outcome measures increases the models’ conditional *R*^2^ by a considerable 12.5% for ADAS-cog, 15.7% for CDRSOB, and 9.3% for MMSE, as can be demonstrated by comparing the best models to the respective reduced models with all terms containing SNE removed (data not shown). This demonstrates that structural connectivity in general and SNE, in particular, may increase the accuracy of predictions of cognitive decline in elderly persons at risk of cognitive decline due to increased amounts of biomarkers of AD-typical pathology. Furthermore, future studies might investigate the factors that determine individual SNE independent of cerebral pathology to develop intervention strategies aimed at improving resilience in individuals.

This study has several limitations. First, SNE and pathology measures were measured at baseline. As such, further longitudinal associations or interactions between them could not be taken into account statistically. This means that the modulation of the association of SNE with resilience to cognitive decline by pathology measures might have another alternative explanation: a lower association of resilience to cognitive decline with SNE at higher baseline pathology could also be explained by a time-lagged deteriorative association between the pathology measure and SNE, whereas in this view a higher association between resilience to cognitive decline with SNE at higher baseline pathology could be explained by time-lagged (compensatory) neuroplasticity of SNE as a response to increasing baseline pathology. Second, the sample considered potentially includes subjects with future sporadic or familial AD, and the MCI cohort has additionally been enriched for memory impairment. As such, the variables considered probably do not follow a distribution representative for the general population, which may limit the generalizability of results. However, cognitive status (CN and MCI) was controlled for as a random variable in statistical modeling. Third, global pathology measures were used. Fourth, data were acquired at different sites and scanners. However, including the center as an additional random effect did not alter results (data not shown).

In conclusion, higher SNE may be associated with lower cognitive decline in cognitively healthy elderly and patients of MCI, especially so at the earlier stages of the AD biomarker cascade characterized by increased amounts of baseline cerebral amyloid load and low amounts of baseline CSF tau. SNE may thus be a surrogate marker for the physiological basis of resilience to cognitive decline and provide a direction for further research aimed at improving the prediction of cognitive decline of persons at risk of dementia as well as research investigating factors leading to higher individual SNE to design intervention strategies aiming to improve individual resilience to cognitive decline.

## Data Availability Statement

Publicly available datasets were analyzed in this study. The data can be accessed at: http://adni.loni.usc.edu/.

## Ethics Statement

The studies involving human participants were reviewed and approved by the local responsible committees on human experimentation at each participating site (institutional and national) and with the Helsinki Declaration of 1975, and the applicable revisions at the time of the investigation. The participating sites with the respective local institutional committees can be found at: https://www.ncbi.nlm.nih.gov/pmc/articles/PMC2927112/pdf/nihms217119.pdf. The patients/participants provided their written informed consent to participate in this study.

## Author Contributions

All authors listed have made a substantial, direct and intellectual contribution to the work, and approved it for publication.

## Conflict of Interest

The authors declare that the research was conducted in the absence of any commercial or financial relationships that could be construed as a potential conflict of interest.

## Footnotes

^1^http://adni.loni.usc.edu

^2^http://adni.loni.usc.edu/methods

^3^http://adni.loni.usc.edu/data-samples/genetic-data

^4^http://www.fil.ion.ucl.ac.uk/spm/

^5^http://dbm.neuro.uni-jena.de/vbm8/

^6^http://adni.loni.usc.edu/methods/pet-analysis/

^7^https://adni.loni.usc.edu/wp-content/uploads/2008/07/adni2-procedures-manual.pdf

## References

[B1] Amlien I. K. Fjell A. M. Walhovd K. B. Selnes P. Stenset V. Grambaite R.. (2013). Mild cognitive impairment: cerebrospinal fluid tau biomarker pathologic levels and longitudinal changes in white matter integrity. Radiology 266, 295–303. 10.1148/radiol.12120319 23151827

[B2] Barulli D. Stern Y. (2013). Efficiency, capacity, compensation, maintenance, plasticity: emerging concepts in cognitive reserve. Trends Cogn. Sci. 17, 502–509. 10.1016/j.tics.2013.08.012 24018144 PMC3840716

[B3] Bates D. Mächler M. Bolker B. Walker S. (2014). Fitting Linear Mixed-Effects Models Using lme4. J. Stat. Soft. 67, 1–57. 10.18637/jss.v067.i01

[B4] Bathelt J. Scerif G. Nobre K. Astle D. E. (2018). Whole-brain white matter organization, intelligence and educational attainment. Trends Neurosci. Educ. 15, 38–47. 10.1016/j.tine.2019.02.004 31176470 PMC6556839

[B5] Blennow K. Vanmechelen E. Hampel H. (2001). CSF total tau, Aβ42 and phosphorylated tau protein as biomarkers for Alzheimer’s disease. Mol. Neurobiol. 24:87. 10.1385/MN:24:1-3:087 11831556

[B6] Cabeza R. Albert M. Belleville S. Craik F. I. M. Duarte A. Grady C. L.. (2018). Maintenance, reserve and compensation: the cognitive neuroscience of healthy ageing. Nat. Rev. Neurosci. 19, 701–710. 10.1038/s41583-018-0068-2 30305711 PMC6472256

[B7] Damoiseaux J. S. (2017). Effects of aging on functional and structural brain connectivity. NeuroImage 160, 32–40. 10.1016/j.neuroimage.2017.01.077 28159687

[B8] Deary I. J. Penke L. Johnson W. (2010). The neuroscience of human intelligence differences. Nat. Rev. Neurosci. 11, 201–211. 10.1038/nrn2793 20145623

[B9] DeCarli C. Fletcher E. Ramey V. Harvey D. Jagust W. J. (2005). Anatomical mapping of white matter hyperintensities (WMH): exploring the relationships between periventricular WMH, deep WMH and total WMH burden. Stroke 36, 50–55. 10.1161/01.STR.0000150668.58689.f2 15576652 PMC3816357

[B10] Donohue M. C. Sperling R. A. Petersen R. Sun C.-K. Weiner M. W. Aisen P. S. (2017). Association between elevated brain amyloid and subsequent cognitive decline among cognitively normal persons. JAMA 317, 2305–2316. 10.1001/jama.2017.6669 28609533 PMC5736301

[B11] Fernández-Cabello S. Valls-Pedret C. Schurz M. Vidal-Piñeiro D. Sala-Llonch R. Bargallo N.. (2016). White matter hyperintensities and cognitive reserve during a working memory task: a functional magnetic resonance imaging study in cognitively normal older adults. Neurobiol. Aging 48, 23–33. 10.1016/j.neurobiolaging.2016.08.008 27636672

[B12] Fischer F. U. Wolf D. Fellgiebel A. (2019). Connectivity and morphology of hubs of the cerebral structural connectome are associated with brain resilience in AD- and age-related pathology. Brain Imaging Behav. 13, 1650–1664. 10.1007/s11682-019-00090-y 30980275

[B13] Fischer F.U. Wolf D. Scheurich A. Fellgiebel A. (2014). Association of structural global brain network properties with intelligence in normal aging. PLoS One 9:e86258. 10.1371/journal.pone.0086258 24465994 PMC3899224

[B14] Fletcher E. Singh B. Harvey D. Carmichael O. DeCarli C. (2012). “Adaptive image segmentation for robust measurement of longitudinal brain tissue change,”. in 2012 Annual International Conference of the IEEE Engineering in Medicine and Biology Society (San Diego, CA: IEEE), 5319–5322. 10.1109/EMBC.2012.6347195 23367130 PMC3776590

[B15] Fornito A. Zalesky A. Breakspear M. (2015). The connectomics of brain disorders. Nat. Rev. Neurosci. 16, 159–172. 10.1038/nrn3901 25697159

[B16] Fox J. Weisberg S. (2019). An R Companion to Applied Regression, 3rd Edition. (Thousand Oaks, CA: SAGE Publications). Available online at: https://socialsciences.mcmaster.ca/jfox/Books/Companion/.

[B17] Greven S. Kneib T. (2010). On the behaviour of marginal and conditional AIC in linear mixed models. Biometrika 97, 773–789. Available online at: https://www.jstor.org/stable/29777136. Accessed 29 January 2021.

[B18] Halekoh U. Højsgaard S. (2014). A Kenward-Roger approximation and parametric bootstrap methods for tests in linear mixed models the RPackage pbkrtest. J. Stat. Softw. 59, 1–32. 10.18637/jss.v059.i0926917999

[B19] Insel P. S. Donohue M. C. Mackin R. S. Aisen P. S. Hansson O. Weiner M. W.. (2016). Cognitive and functional changes associated with A? pathology and the progression to mild cognitive impairment. Neurobiol. Aging. 48, 172–181. 10.1016/j.neurobiolaging.2016.08.017 27710807

[B20] Jack C. R. Knopman D. S. Jagust W. J. Petersen R. C. Weiner M. W. Aisen P. S.. (2013). Tracking pathophysiological processes in Alzheimer’s disease: an updated hypothetical model of dynamic biomarkers. Lancet Neurol. 12, 207–216. 10.1016/S1474-4422(12)70291-0 23332364 PMC3622225

[B21] Jack C. R. Therneau T. M. Wiste H. J. Weigand S. D. Knopman D. S. Lowe V. J.. (2016). Transition rates between amyloid and neurodegeneration biomarker states and to dementia: a population-based, longitudinal cohort study. Lancet Neurol. 15, 56–64. 10.1016/S1474-4422(15)00323-3 26597325 PMC4784263

[B22] Karran E. Mercken M. Strooper B. D. (2011). The amyloid cascade hypothesis for Alzheimer’s disease: an appraisal for the development of therapeutics. Nat. Rev. Drug. Discov. 10, 698–712. 10.1038/nrd3505 21852788

[B23] Kljajevic V. Grothe M. J. Ewers M. Teipel S. (2014). Distinct pattern of hypometabolism and atrophy in preclinical and predementia Alzheimer’s disease. Neurobiol. Aging 35, 1973–1981. 10.1016/j.neurobiolaging.2014.04.006 24811241

[B24] Koenis M. M. G. Brouwer R. M. Swagerman S. C. van Soelen I. L. C. Boomsma D. I. Pol H. E. H. (2018). Association between structural brain network efficiency and intelligence increases during adolescence. Hum. Brain Mapp. 39, 822–836. 10.1002/hbm.23885 29139172 PMC6866576

[B25] Koller M. (2016). Robustlmm: an R package for robust estimation of linear mixed-effects models. J. Stat .Softw. 75:i06. 10.18637/jss.v075.i06 PMC7351245 32655332

[B26] Latora V. Marchiori M. (2001). Efficient behavior of small-world networks. Phys. Rev. Lett. 87:198701. 10.1103/PhysRevLett.87.198701 11690461

[B27] Li Y. Liu Y. Li J. Qin W. Li K. Yu C.. (2009). Brain anatomical network and intelligence. PLoS Comput. Biol. 5:e1000395. 10.1371/journal.pcbi.1000395 19492086 PMC2683575

[B28] Nakagawa S. Schielzeth H. (2013). A general and simple method for obtaining R2 from generalized linear mixed-effects models. Methods. Ecol. Evol. 4, 133–142. 10.1093/sysbio/syy060 30239975

[B29] Penke L. Maniega S. M. Bastin M. E. Valdés Hernández M. C. Murray C. Royle N. A.. (2012). Brain white matter tract integrity as a neural foundation for general intelligence. Mol. Psychiatry 17, 1026–1030. 10.1038/mp.2012.66 22614288

[B30] Reuter-Lorenz P. A. Park D. C. (2014). How does it STAC Up? Revisiting the scaffolding theory of aging and cognition. Neuropsychol Rev. 24, 355–370. 10.1007/s11065-014-9270-9 25143069 PMC4150993

[B31] Rohde G. K. Barnett A. S. Basser P. J. Marenco S. Pierpaoli C. (2004). Comprehensive approach for correction of motion and distortion in diffusion-weighted MRI. Magn. Reson. Med. 51, 103–114. 10.1002/mrm.10677 14705050

[B32] Rosen W. G. Mohs R. C. Davis K. L. (1984). A new rating scale for Alzheimer’s disease. Am. J. Psychiatry 141, 1356–1364. 10.1176/ajp.141.11.1356 6496779

[B33] Säfken B. Rügamer D. Kneib T. Greven S. (2018). Conditional model selection in mixed-effects models with cAIC4. ArXiv [Preprint]. Available online at: http://arxiv.org/abs/1803.05664.

[B34] Schmand B. Smit J. H. Geerlings M. I. Lindeboom J. (1997). The effects of intelligence and education on the development of dementia. A test of the brain reserve hypothesis. Psychol Med. 27, 1337–1344. 10.1017/s0033291797005461 9403905

[B35] Sebastian A. Baldermann C. Feige B. Katzev M. Scheller E. Hellwig B.. (2013). Differential effects of age on subcomponents of response inhibition. Neurobiol. Aging. 34, 2183–2193. 10.1016/j.neurobiolaging.2013.03.013 23591131

[B36] Shaw L. M. Vanderstichele H. Knapik-Czajka M. Clark C. M. Aisen P. S. Petersen R. C.. (2009). Cerebrospinal fluid biomarker signature in Alzheimer’s disease neuroimaging initiative subjects. Ann. Neurol. 65, 403–413. 10.1002/ana.21610 19296504 PMC2696350

[B37] Skillbäck T. Farahmand B. Y. Rosén C. Mattsson N. Nägga K. Kilander L.. (2015). Cerebrospinal fluid tau and amyloid 1-42 in patients with dementia. Brain 138, 2716–2731. 10.1093/brain/awv181 26133663

[B38] Smith R. E. Tournier J. D. Calamante F. Connelly A. (2012). Anatomically-constrained tractography: improved diffusion MRI streamlines tractography through effective use of anatomical information. NeuroImage. 62, 1924–1938. 10.1016/j.neuroimage.2012.06.005 22705374

[B39] Solé-Padullés C. Lladó A. Bartrés-Faz D. Fortea J. Sánchez-Valle R. Bosch B.. (2011). Association between cerebrospinal fluid tau and brain atrophy is not related to clinical severity in the Alzheimer’s disease continuum. Psychiatry Res. Neuroimaging 192, 140–146. 10.1016/j.pscychresns.2010.12.001 21546220

[B40] Stargardt A. Swaab D. F. Bossers K. (2015). The storm before the quiet: neuronal hyperactivity and Aβ in the presymptomatic stages of Alzheimer’s disease. Neurobiol. Aging 36, 1–11. 10.1016/j.neurobiolaging.2014.08.014 25444609

[B41] Stern Y. (2012). Cognitive reserve in ageing and Alzheimer’s disease. Lancet Neurol. 11, 1006–1012. 10.1016/S1474-4422(12)70191-6 23079557 PMC3507991

[B42] Tsvetanov K. A. Henson R. N. A. Tyler L. K. Razi A. Geerligs L. Ham T. E.. (2016). Extrinsic and intrinsic brain network connectivity maintains cognition across the lifespan despite accelerated decay of regional brain activation. J. Neurosci. 36, 3115–3126. 10.1523/JNEUROSCI.2733-15.2016 26985024 PMC4792930

[B43] Tsvetanov K. A. Ye Z. Hughes L. Samu D. Treder M. S. Wolpe N.. (2018). Activity and connectivity differences underlying inhibitory control across the adult life span. J. Neurosci. 38, 7887–7900. 10.1523/JNEUROSCI.2919-17.2018 30049889 PMC6125816

[B44] Vernooij M. W. Ikram M. A. Vrooman H. A. Wielopolski P. A. Krestin G. P. Hofman A.. (2009). White matter microstructural integrity and cognitive function in a general elderly population. Arch. Gen. Psychiatry 66, 545–553. 10.1001/archgenpsychiatry.2009.5 19414714

[B45] Vos S. J. Xiong C. Visser P. J. Jasielec M. S. Hassenstab J. Grant E. A.. (2013). Preclinical Alzheimer’s disease and its outcome: a longitudinal cohort study. Lancet Neurol. 12, 957–965. 10.1016/S1474-4422(13)70194-7 24012374 PMC3904678

[B46] Whalley L. J. Deary I. J. Appleton C. L. Starr J. M. (2004). Cognitive reserve and the neurobiology of cognitive aging. Ageing Res. Rev. 3, 369–382. 10.1016/j.arr.2004.05.001 15541707

[B47] Wolf D. Fischer F. U. Fellgiebel A. (2018). A methodological approach to studying resilience mechanisms: demonstration of utility in age and Alzheimer’s disease-related brain pathology. Brain Imaging Behav. 1, 1–10. 10.1007/s11682-018-9870-8 29713998

[B48] Wook Yoo S. Han C. E. Shin J. S. Seo W. S. Na D. L. Kaiser M.. (2015). A network flow-based analysis of cognitive reserve in normal ageing and Alzheimer’s disease. Sci. Rep. 5:10057. 10.1038/srep10057 25992968 PMC4438712

[B49] Yaffe K. Weston A. Graff-Radford N. R. Satterfield S. Simonsick E. M. Younkin S. G.. (2011). Association of plasma β-Amyloid level and cognitive reserve with subsequent cognitive decline. JAMA 305, 261–266. 10.1001/jama.2010.1995 21245181 PMC3108075

