# Supplementary material for: Structural Network Efficiency Predicts Resilience to Cognitive Decline in Elderly at Risk for Alzheimer’s Disease
Source: Front Aging Neurosci. 2021 Feb 22;13:637002. doi: 10.3389/fnagi.2021.637002 (PMC7937862; doi:10.3389/fnagi.2021.637002)
Supplement: Supplementary file 1 [file Table_1.DOCX]

**Supplementary Material**

**Description of the statistical approach to investigate resilience**

Any analysis of resilience requires at least three types of measures: a possible resilience factor, a measure of pathology and a measure of cognitive outcome (Stern, 2012). However, analyses that include these three measures in regression analyses traditionally only consider a positive interaction between pathology and the possible resilience factor as evidence of resilience (Craik *et al.*, 2011). Recently, we published a reconceptualization of this approach that aims to operationalize a differentiation of resilience by its relation to pathology (Wolf *et al.*, 2018). The investigation of resilience in the present paper is based on an extension of this approach.

Specifically, we model resilience as cognitive outcome not explained by pathology measures (see table 2 for an overview of pathology measures). A candidate resilience factor could then be associated with resilience in three ways. First, the association between resilience factor and resilience can be unmodulated by pathology measures, which requires that the mechanism behind the association of the resilience factor and resilience remains intact even in the presence of increased pathology. This is referred to as *general resilience*. Second, the association between resilience factor and resilience could be positively modulated by one or more pathology measures, indicating that the importance of the resilience factor for better cognition than predicted by pathology increases with increasing amounts of the respective pathology. This is termed *dynamical resilience*. Third, a resilience factor for which general or dynamic resilience has been established may additionally show an association with resilience that is negatively modulated by a pathology measure. This indicates that while the resilience factor is associated with resilience in certain scenarios – either on average with respect to the considered pathology measure or dynamically with respect to another pathology measure – the association with resilience diminishes with increasing amounts of the respective pathology measure. This is termed *limited resilience*.

These concepts can be statistically implemented using the following model equation:

COG ~ RES + PATH_1_ + PATH_2_ + … + PATH_n_ + RES * (PATH_1_ + PATH_2_ + … +

PATH_n_)

where COG represents the cognitive outcome measure, PATH represents a measure of pathology, n is the number of pathology measures considered and RES represents a possible resilience factor. Note that as PATH is partialled out of all other variables by the regression estimation, COG as well as RES represent the variance of these measures unexplained by PATH. For this reason COG is referred to as resilience (Wolf *et al.*, 2018).

This model supports the three types of resilience in the following cases:

**General resilience** – the term RES is positively associated with COG.

**Dynamic resilience** – one or more of the terms RES * PATH are positively associated with COG. RES can be positively associated with COG as well, in this case one could add that *on average* of PATH, RES is positively associated with COG.

**Limited resilence** – either RES and/or one or more of the terms RES * PATH are positively associated with COG (i.e. general or dynamic resilience). In addition one or more of the remaining (i.e. different) terms RES * PATH are negatively associated with COG.

Finally, the concepts of general and dynamic resilience overlap but are not equivalent to the concept of brain reserve and cognitive reserve put forward by(Barulli and Stern, 2013): whereas the definitions of brain reserve and cognitive reserve refer to the quality of the underlying mechanism of resilience, the concept of general and dynamic resilience is based entirely on the empirical manifestation of resilience as associations with the resilience factor (Wolf *et al.*, 2018). Brain reserve could thus manifest as general resilience or dynamic resilience (e.g. dynamic in the case of a threshold based association), as might cognitive reserve (which may have a positive effect on cognition even in the absence of pathology).

**Model adaption to longitudinal data**
As the aim of the present study is to investigate resilience to cognitive decline, i.e. lower cognitive decline than expected based on baseline pathology measures, the base resilience model described in the previous section needs to be extended. The original model (with all pathology measures collapsed into PATH for readability) is given as

   COG ~ RES * PATH + RES + PATH.

To accommodate longitudinal data and model the resilience to cognitive decline, this model is extended by the addition of a time variable T as well as its interaction terms with the other independent variables:

COG ~ (RES * PATH + RES + PATH) * T + T

Note that the effect of PATH as well as the modulation of the association of the T with COG by PATH, i.e. cognitive decline due to baseline pathology, are partialled out of COG. In analogy to the original model, this extended model supports resilience to cognitive decline in the following scenarios:

**General resilience to cognitive decline** – the term RES * T is positively associated with COG.

**Dynamic resilience to cognitive decline** – one or more of the terms RES * T * PATH are positively associated with COG. RES * T can be positively associated with COG as well, in this case one could add that *on average* of PATH, RES * T is associated with COG.

**Limited resilence to cognitive decline** – either RES * T and/or one or more of the terms RES * T * PATH are positively associated with COG (i.e. general or dynamic resilience to cognitive decline). In addition one or more of the remaining (i.e. different) terms RES * T * PATH are negatively associated with COG.

**Statistical analysis**

Longitudinal ADAS-cog, CDRSOB and MMSE measures were coded in single variables and set as cognitive outcome and thus as the dependent variables. The time of assessment of the cognitive outcome variables was encoded as an additional time variable that was set as an independent variable. Other independent variables consisted of baseline SNE as potential resilience factor as well as AV45, TAU and WMHV as measures of baseline pathology (see table 2 for descriptive statistics). For each of these, a family of models were estimated representing all possible combinations of the following terms: SNE, the interaction term of SNE and the time variable (general resilience), the first order interaction terms of SNE and pathology measures as well as the second order interaction terms of SNE, the time variable and pathology measures (dynamic or limited resilience). Note that for models including interaction terms, the constituent terms were also always included in the models - including all three possible first order interaction terms of the individual elements of second order interaction terms. In total, this yielded 36 models to be estimated for each cognitive outcome measure. For a list containing the terms of each model, please refer to the section below.

The models were estimated as linear mixed effects models, wherein random intercepts for each subject and for clinical status at baseline (CN/MCI) were estimated. Additionally, all models included the baseline pathology measures AV45, TAU and WMHV as well as their interaction terms with the time variable. Years of education, age, gender and APOE4 positivity were included in all models as covariates. Subsequently, for each cognitive outcome measure and the corresponding family of 36 possible resilience models, the conditional Akaike Information Criterion (cAIC)was calculated (Greven and Kneib, 2010) as well as the Delta cAIC scores (differences between the model with the lowest cAIC score and each of the remaining models), the Akaike weights and the marginal coefficient of determination. All models with Delta cAIC lower than 2 were considered as models with substantial evidence.
The model terms of interest as per the extended resilience model described in the previous section were the interaction term between the time variable and SNE (**general resilience** to cognitive decline) as well as the second order interaction terms of the time variable with SNE and with baseline pathology measures (**dynamic or limited resilience** to cognitive decline). For these terms, significance testing was carried out if they were included in all models with Delta cAIC lower than 2 for each cognitive outcome. Significance testing was conducted using the LRT test as well as parametric bootstrapping at 1 million simulations per test. In the case of dynamic or limited resilience, the model with the lowest cAIC was compared to a reduced model with the term of interest (*t * SNE * path*) removed. In the case of general resilience to cognitive decline, different models had to be compared, as the term of interest (*t * SNE*) is a constituent term of all second order interaction terms (including t * SNE * path). For this reason, all second order interaction terms were removed from the model with the lowest cAIC. This model was then compared to a further reduced model with the term of interest removed. Furthermore, the models with lowest cAIC were reestimated using robust linear mixed regression models.

All statistical analyses were conducted using R 3.4.0 as well as the packages ”lme4“ (Bates *et al.*, 2014) ”robustlmm“ (Koller, 2016), ”cAIC4” (Säfken *et al.*, 2018), ”pbkrtest” (Halekoh and Højsgaard, 2014) as well as ”car” (Fox and Weisberg, 2019). For cAIC and significance testing, models were estimated with maximum likelihood estimation. For coefficient reporting, models were reestimated using restricted maximum likelihood estimation. Two data points with implausibly low MMSE measurements (4 and 6) were removed in the respective models. All variables that were part of an interaction term in any model were centered to reduce collinearity (Aiken *et al.*, 1991). Variance inflation was calculated for the best model for each cognitive outcome variable. WMHV was log-transformed to achieve approximate normal distribution. The significance threshold was set to α = 0.05 for all analyses. Results were corrected for FDR at 5%.

**List of the family of models considered and evaluated**

The following list generally only contains the present highest order interaction terms for brevity, their constituent terms were always included; SNE: white matter network efficiency, WMHV: white matter hyperintensity volume.

[1] "SNE + T + AV45 + TAU + WMHV + Age + Gender + APOE4 + Education + SNE*T + SNE*AV45 + T*AV45 + SNE*TAU + T*TAU + T*WMHV + SNE*T*AV45 + (1|Subject) + (1|Diagnosis)"

[2] "SNE + T + AV45 + WMHV + TAU + Age + Gender + APOE4 + Education + SNE*T + SNE*AV45 + T*AV45 + SNE*WMHV + T*TAU + T*WMHV + SNE*T*AV45 + (1|Subject) + (1|Diagnosis)"

[3] "SNE + T + AV45 + TAU + WMHV + Age + Gender + APOE4 + Education + SNE*T + SNE*AV45 + T*AV45 + SNE*TAU + SNE*WMHV + T*TAU + T*WMHV + SNE*T*AV45 + (1|Subject) + (1|Diagnosis)"

[4] "SNE + T + TAU + AV45 + WMHV + Age + Gender + APOE4 + Education + SNE*T + SNE*TAU + T*TAU + SNE*AV45 + T*AV45 + T*WMHV + SNE*T*TAU + (1|Subject) + (1|Diagnosis)"

[5] "SNE + T + TAU + WMHV + AV45 + Age + Gender + APOE4 + Education + SNE*T + SNE*TAU + T*TAU + SNE*WMHV + T*AV45 + T*WMHV + SNE*T*TAU + (1|Subject) + (1|Diagnosis)"

[6] "SNE + T + TAU + AV45 + WMHV + Age + Gender + APOE4 + Education + SNE*T + SNE*TAU + T*TAU + SNE*AV45 + SNE*WMHV + T*AV45 + T*WMHV + SNE*T*TAU + (1|Subject) + (1|Diagnosis)"

[7] "SNE + T + WMHV + AV45 + TAU + Age + Gender + APOE4 + Education + SNE*T + SNE*WMHV + T*WMHV + SNE*AV45 + T*AV45 + T*TAU + SNE*T*WMHV + (1|Subject) + (1|Diagnosis)"

[8] "SNE + T + WMHV + TAU + AV45 + Age + Gender + APOE4 + Education + SNE*T + SNE*WMHV + T*WMHV + SNE*TAU + T*AV45 + T*TAU + SNE*T*WMHV + (1|Subject) + (1|Diagnosis)"

[9] "SNE + T + WMHV + AV45 + TAU + Age + Gender + APOE4 + Education + SNE*T + SNE*WMHV + T*WMHV + SNE*AV45 + SNE*TAU + T*AV45 + T*TAU + SNE*T*WMHV + (1|Subject) + (1|Diagnosis)"

[10] "SNE + T + AV45 + TAU + WMHV + Age + Gender + APOE4 + Education + SNE*T + SNE*AV45 + T*AV45 + SNE*TAU + T*TAU + SNE*WMHV + T*WMHV + SNE*T*AV45 + SNE*T*TAU + (1|Subject) + (1|Diagnosis)"

[11] "SNE + T + AV45 + WMHV + TAU + Age + Gender + APOE4 + Education + SNE*T + SNE*AV45 + T*AV45 + SNE*WMHV + T*WMHV + SNE*TAU + T*TAU + SNE*T*AV45 + SNE*T*WMHV + (1|Subject) + (1|Diagnosis)"

[12] "SNE + T + TAU + WMHV + AV45 + Age + Gender + APOE4 + Education + SNE*T + SNE*TAU + T*TAU + SNE*WMHV + T*WMHV + SNE*AV45 + T*AV45 + SNE*T*TAU + SNE*T*WMHV + (1|Subject) + (1|Diagnosis)"

[13] "SNE + AV45 + T + TAU + WMHV + Age + Gender + APOE4 + Education + SNE*AV45 + AV45*T + T*TAU + T*WMHV + (1|Subject) + (1|Diagnosis)"

[14] "SNE + TAU + T + AV45 + WMHV + Age + Gender + APOE4 + Education + SNE*TAU + T*AV45 + TAU*T + T*WMHV + (1|Subject) + (1|Diagnosis)"

[15] "SNE + T + AV45 + TAU + WMHV + Age + Gender + APOE4 + Education + SNE*T + T*AV45 + T*TAU + T*WMHV + (1|Subject) + (1|Diagnosis)"

[16] "SNE + WMHV + T + AV45 + TAU + Age + Gender + APOE4 + Education + SNE*WMHV + T*AV45 + T*TAU + WMHV*T + (1|Subject) + (1|Diagnosis)"

[17] "SNE + AV45 + TAU + T + WMHV + Age + Gender + APOE4 + Education + SNE*AV45 + SNE*TAU + AV45*T + TAU*T + T*WMHV + (1|Subject) + (1|Diagnosis)"

[18] "SNE + AV45 + T + TAU + WMHV + Age + Gender + APOE4 + Education + SNE*AV45 + SNE*T + AV45*T + T*TAU + T*WMHV + (1|Subject) + (1|Diagnosis)"

[19] "SNE + AV45 + WMHV + T + TAU + Age + Gender + APOE4 + Education + SNE*AV45 + SNE*WMHV + AV45*T + T*TAU + WMHV*T + (1|Subject) + (1|Diagnosis)"

[20] "SNE + TAU + T + AV45 + WMHV + Age + Gender + APOE4 + Education + SNE*TAU + SNE*T + T*AV45 + TAU*T + T*WMHV + (1|Subject) + (1|Diagnosis)"

[21] "SNE + TAU + WMHV + T + AV45 + Age + Gender + APOE4 + Education + SNE*TAU + SNE*WMHV + T*AV45 + TAU*T + WMHV*T + (1|Subject) + (1|Diagnosis)"

[22] "SNE + T + WMHV + AV45 + TAU + Age + Gender + APOE4 + Education + SNE*T + SNE*WMHV + T*AV45 + T*TAU + T*WMHV + (1|Subject) + (1|Diagnosis)"

[23] "SNE + AV45 + TAU + T + WMHV + Age + Gender + APOE4 + Education + SNE*AV45 + SNE*TAU + SNE*T + AV45*T + TAU*T + T*WMHV + (1|Subject) + (1|Diagnosis)"

[24] "SNE + AV45 + TAU + WMHV + T + Age + Gender + APOE4 + Education + SNE*AV45 + SNE*TAU + SNE*WMHV + AV45*T + TAU*T + WMHV*T + (1|Subject) + (1|Diagnosis)"

[25] "SNE + AV45 + T + WMHV + TAU + Age + Gender + APOE4 + Education + SNE*AV45 + SNE*T + SNE*WMHV + AV45*T + T*TAU + T*WMHV + (1|Subject) + (1|Diagnosis)"

[26] "SNE + TAU + T + WMHV + AV45 + Age + Gender + APOE4 + Education + SNE*TAU + SNE*T + SNE*WMHV + T*AV45 + TAU*T + T*WMHV + (1|Subject) + (1|Diagnosis)"

[27] "SNE + AV45 + TAU + T + WMHV + Age + Gender + APOE4 + Education + SNE*AV45 + SNE*TAU + SNE*T + SNE*WMHV + AV45*T + TAU*T + T*WMHV + (1|Subject) + (1|Diagnosis)"

[28] "SNE + T + AV45 + TAU + WMHV + Age + Gender + APOE4 + Education + SNE*T + SNE*AV45 + T*AV45 + T*TAU + T*WMHV + SNE*T*AV45 + (1|Subject) + (1|Diagnosis)"

[29] "SNE + T + TAU + AV45 + WMHV + Age + Gender + APOE4 + Education + SNE*T + SNE*TAU + T*TAU + T*AV45 + T*WMHV + SNE*T*TAU + (1|Subject) + (1|Diagnosis)"

[30] "SNE + T + WMHV + AV45 + TAU + Age + Gender + APOE4 + Education + SNE*T + SNE*WMHV + T*WMHV + T*AV45 + T*TAU + SNE*T*WMHV + (1|Subject) + (1|Diagnosis)"

[31] "SNE + T + AV45 + TAU + WMHV + Age + Gender + APOE4 + Education + SNE*T + SNE*AV45 + T*AV45 + SNE*TAU + T*TAU + T*WMHV + SNE*T*AV45 + SNE*T*TAU + (1|Subject) + (1|Diagnosis)"

[32] "SNE + T + AV45 + WMHV + TAU + Age + Gender + APOE4 + Education + SNE*T + SNE*AV45 + T*AV45 + SNE*WMHV + T*WMHV + T*TAU + SNE*T*AV45 + SNE*T*WMHV + (1|Subject) + (1|Diagnosis)"

[33] "SNE + T + TAU + WMHV + AV45 + Age + Gender + APOE4 + Education + SNE*T + SNE*TAU + T*TAU + SNE*WMHV + T*WMHV + T*AV45 + SNE*T*TAU + SNE*T*WMHV + (1|Subject) + (1|Diagnosis)"

[34] "SNE + T + AV45 + TAU + WMHV + Age + Gender + APOE4 + Education + SNE*T + SNE*AV45 + T*AV45 + SNE*TAU + T*TAU + SNE*WMHV + T*WMHV + SNE*T*AV45 + SNE*T*TAU + SNE*T*WMHV + (1|Subject) + (1|Diagnosis)"

[35] "SNE + T + AV45 + TAU + WMHV + Age + Gender + APOE4 + Education + T*AV45 + T*TAU + T*WMHV + (1|Subject) + (1|Diagnosis)"

[36] "T + AV45 + TAU + WMHV + Age + Gender + APOE4 + Education + T*AV45 + T*TAU + T*WMHV + (1|Subject) + (1|Diagnosis)"

***Table: Overview and comparison of best fitting models.*** *Cog Out, cognitive outcome variable. No. Par, number of estimated parameters. Log(L), log-likelihood. cAIC, conditional Akaike information criterion. ΔcAIC, difference of the cAIC of the model considered and the model with lowest cAIC. ωcAIC, Akaike weights. R²m, marginal coefficient of determination. ADAS-cog, Alzheimer’s disease assessment scale. CDRSOB, clinical dementia rating sum of boxes. MMSE, minimental state examination.*

| Cog Out | Model | No. Par | log(L) | cAIC | ΔcAIC | ωcAIC | R²m |
| --- | --- | --- | --- | --- | --- | --- | --- |
| ADAS-cog | 10 | 19 | -1248.1 | 2388.5 | 0.3266 | .2803 | .4045 |
| ADAS-cog | 31 | 18 | -1248.6 | 2388.2 | 0 | .3300 | .4120 |
| ADAS-cog | 34 | 20 | -1247.9 | 2389.6 | 1.4286 | .1615 | .4053 |
| CDRSOB | 34 | 20 | -580.1 | 1089.5 | 0 | .6528 | .4333 |
| MMSE | 10 | 19 | -791.3 | 1523.3 | 0.2682 | .2177 | .4092 |
| MMSE | 31 | 18 | -791.5 | 1523.1 | 0 | .2489 | .4155 |

**References**

Barulli, D., & Stern, Y. (2013). Efficiency, capacity, compensation, maintenance, plasticity: emerging concepts in cognitive reserve. *Trends in Cognitive Sciences*, *17*(10), 502–509.

Bates D, Mächler M, Bolker B, Walker S. Fitting Linear Mixed-Effects Models using lme4 [Internet]. 2014[cited 2018 Sep 21] Available from: https://arxiv.org/abs/1406.5823

Craik, F. I. M., Salthouse, T. A., & Salthouse, T. A. (2011, March 15). Intelligence, Education, and the Brain Reserve Hypothesis: Helen Christensen, Kaarin J. Anstey, Liana S. Leach, and Andrew J. Mackinnon. *The Handbook of Aging and Cognition*. Retrieved September 20, 2018, from https://www.taylorfrancis.com/

Fox J, Weisberg S. An R Companion to Applied Regression [Internet]. Third. Sage; 2019Available from: https://socialsciences.mcmaster.ca/jfox/Books/Companion/

Greven S, Kneib T. On the behaviour of marginal and conditional AIC in linear mixed models. Biometrika 2010; 97: 773–89.

Halekoh U, Højsgaard S. A Kenward-Roger Approximation and Parametric Bootstrap Methods for Tests in Linear Mixed Models - The *R* Package **pbkrtest** [Internet]. J Stat Softw 2014; 59[cited 2020 Jan 31] Available from: http://www.jstatsoft.org/v59/i09/

Koller M. robustlmm: An R Package for Robust Estimation of Linear Mixed-Effects Models [Internet]. J Stat Softw 2016[cited 2018 Sep 20] Available from: https://www.jstatsoft.org/article/view/v075i06

Säfken B, Rügamer D, Kneib T, Greven S. Conditional Model Selection in Mixed-Effects Models with cAIC4 [Internet]. ArXiv180305664 Stat 2018[cited 2020 Jan 31] Available from: http://arxiv.org/abs/1803.05664

Stern, Y. (2012). Cognitive reserve in ageing and Alzheimer’s disease. *The Lancet Neurology*, *11*(11), 1006–1012.

Wolf, D., Fischer, F. U., & Fellgiebel, A. (2018). A methodological approach to studying resilience mechanisms: demonstration of utility in age and Alzheimer’s disease-related brain pathology. *Brain Imaging and Behavior*, 1–10.
